# Longevity and Economic Growth in China and India Using a Newly Developed Bootstrap ARDL Model

**DOI:** 10.3389/fpubh.2020.00291

**Published:** 2020-08-07

**Authors:** Feng Li Lin, Mei-Chih Wang

**Affiliations:** ^1^Department of Accounting, Chaoyang University of Technology, Taichung, Taiwan; ^2^Department of Finance, College of Management, Providence University, Taichung, Taiwan

**Keywords:** longevity, economic growth, granger causality test, bootstrap ARDL model, alcohol consumption, J1, N3, I1, J6

## Abstract

In this study, we use a recently developed Bootstrap ARDL model to examine the influence of longevity (life expectancy after giving birth) and alcohol consumption on economic progression (GDP) in both China and India during the years between 1992 and 2015. Empirical results have shown an extended link across economic development, longevity, and alcohol use in both China and India. The Granger causality test, derived from the Bootstrap ARDL model, demonstrates a unidirectional relationship between economic growth and longevity in China. However, a bidirectional causality exists between longevity and alcohol use in India. Results have important implications for Indian and Chinese governments' public health policies, focused on alcohol consumption reduction specifically, and population health generally.

## Introduction

Many studies have recently explored the relationship between longevity (life-expectancy) and economic growth. However, few have focused on a causal link between longevity and economic growth. Causal linkage between longevity and economic growth is key for policymakers designing national health policies that promote a long-life. Considering the public health data, the question remains as to why people live longer than ever before? One possible reason, reported by previous studies, is that improved medical health leads to a long-life that supports economic growth. However, we can consider the causality runs in reverse—economic growth improves health. Against this backdrop, the current study revisits the impact of longevity on economic growth (GDP) in both China and India, between the years of 1992 and 2015, using a recently developed Bootstrap ARDL model.

Over half of income inequality, between developed and developing countries, is associated with health and results in a short life expectancy (1). Physical wellness across countries is an insight to societal success (2). Citizens who have experienced war know how rapidly health and wealth can deteriorate (3). In the absence of such experience, citizens must be educated to appreciate the link between health and economic income.

Economic growth is pro-cyclical for health progress, raising life expectancy and lowering mortality, as reported by Pritchett and Summers (4), Filmer and Pritchett (5), Gerdtham and Johannesson (6), Svensson (7), Baird et al. (8), Weil (9), Sharma (10), and Cole (11). In contrast, some studies show a link between economic growth and mortality, a counter-cyclical relationship as reported by Ruhm (12, 13), Cutler et al. (14), Economou et al. (15), Granados (16), van den Berg et al. (17).

Other studies, such as those by Dehejia et al. (18) and Neumayer (19), suggest economic recession is pro-cyclical in relation to health. They assert that during economic recession, the population health improves. Other researchers, such as Ruhm (12, 20), Brenner (21), Granados (22), Granados and Ionides (23), and Chen (24) assert that there is a variable relationship between health progress and business cycles. This perspective is made more complex as longevity exhibits varying results at different stages of the business cycle.

To better understand this complex relationship, we employ information from the World Bank, focusing on life expectancy in the two leading population nations of India and China. India's life expectancy has increased from 41.17 to 68.80 years, while China' has risen from 43.72 to 76.41 years. Both countries' GDP have increased from 47.21 and 42.16 billion USD, in 1962, to 12,237.70 and 2,650.73 billion USD, in 2017, for China and India, respectively. The difference in longevity between the two nations is now 7.61 years, and although GDP started at a comparable level, China's is now 4.62 times larger. Despite having World Class hospitals and medical practitioners, India experiences hygiene and medical education deficiencies when compared with China.

According to the United Nations, China's population will expand until the year 2025. China's total birth and fertility rate has diminished swiftly since the 1970's, due to government policy, resulting in a changing age structure now over weighted to the elderly. In 2000, China became an aging society, with the proportion of the elderly population exceeding 7% for the first time (25). Meanwhile, the population in India is expected to overtake China by 2020 and further increase to over 1.7 billion by the year 2065. This similar starting base qualifies this research frame when studying a health issue common to both countries, i.e., alcohol consumption.

Common determinants of suicidal behavior include depression and alcohol use. Suicide risk rises 7% for people that are dependent on alcohol. Alcohol and other substance use are involved in 25–50% of all suicides (26); and in all deaths from suicide, 22% can result from the use of alcohol, i.e., every fifth suicide could be avoided if alcohol was not consumed in the population (1). Alcohol per capita consumption has risen from 5.5 L in 2005 to 6.4 L in 2010, a trend that varies by region.

South-East Asian regions, such as China and India, exhibit high alcohol consumption (4.1, 7.1, and 7.2 liters in 2005, 2010, and 2016 in China, respectively, and 2.4, 4.3, and 5.7 L in 2005, 2010, and 2016 in India, respectively). The highest increase in alcohol consumption, by 2025, is expected in the South-East Asia Region, with India increasing by 2.2 L and China by 0.9 liters per capita (27). Understanding the causal association in alcohol consumption, income, and health for China and India is vital to health policymakers in in these countries and around the world.

The bootstrap autoregressive-distributed lag (Bootstrap ARDL) test of the cointegration method by McNown et al. (28) was adopted for the current study in order to further elaborate the Granger link between longevity and economic expansion in both China and India, by making use of four different causal hypotheses, including feedback, income, and health perspectives (29, 30). We also employed the neutrality hypothesis (6, 7, 31). Alcohol consumption, income, and health are the relevant variables (9, 22, 29). This model will analyze the three-way relationship among the variables of GDP per capita, longevity, and alcohol consumption in China and India from 1992 to 2015. Previous studies commonly use methodologies such as the Vector Error Correction model (VECM), our study will be the first to employ the Bootstrap ARDL model to investigate this issue, which can overcome the limitation of a small sample size.

The remainder of this paper is organized as follows: Section Theoretical Background and Literature Review describes the literature review and theoretical background. Section Data and Methodology presents the data used in this study, while Section Empirical Results and Policy Implications describes the Granger causality test results using a Bootstrap ARDL model along with policy implications of our findings. Section Conclusions concludes the paper.

## Theoretical Background and Literature Review

We can define our empirical model based on the neoclassic growth model of Zhang et al. (32) defined as follows:

(1) $$\begin{array}{l} \text{y}=\left(\text{x},\text{ z}\right) \end{array}$$

After some manipulation that we can write as the following log-linear form:

(2) $$\begin{array}{l} {\text{y}}_{\text{t}}=\alpha +\beta x_{t}+\gamma z_{t}+\varepsilon_{t} \end{array}$$

Where y is economic growth rate, x is longevity (life-expectancy), and z is a control variable. We use alcohol consumption as a control variable as alcohol consumption affected health and economic growth in both countries, in turn impacting both longevity and economic growth.

Previous studies demonstrate four causality paths between economic development and health. The income view is that increasing incomes will result in an improved overall population health. Theoretically, economic growth can provide governments with the necessary tools to build an improved health system and to invest in technologies, governance, education, and institutional quality. Many researchers, such as Pritchett and Summers (4), Filmer and Pritchett (5), Gerdtham and Johannesson (6), Svensson (7), Baird et al. (8), Weil (9), Sharma (10), Cole (11) show that economic growth is pro-cyclical for health progress.

With the fixed effects Panel data model (from 1960 to 1985), focused on developing countries' child fatality rates, Pritchett and Summers (4) found that the link between mortality rates and GDP per capita is significantly negative, i.e., underprivileged communities in 1990 lost about −0.2%, and −0.4% in the long-term, for every 1% increase in GDP. Filmer and Pritchett (5) utilized cross-national data to find the importance of education in understanding infant fatality in developing countries in the 1990's. As much as 95% of the multi-national variability in mortality can be attributed to ethnic fragmentation, female education, income inequality, income per capita, and predominant religion.

Gerdtham and Johannesson (6) evaluated the impact of business cycles on mortality risk using over 40,000 individuals in Sweden, finding a countercyclical linkage between mortality rate for men and the business cycle. Svensson (7) revealed a counter-cyclical of the business cycle and mortality for those between 20 and 49 years old in 21 Swedish regions, from 1987 to 2003. Using a big data set of 1.7 million deliveries grounded on and without specific trends relating to countries in GDP per capita and infant mortality in 59 underprivileged countries, to examine if immediate variation in GDP can impact mortality from late 1980's to early 2000's.

Baird et al. (8) found an opposite connection between GDP and infant mortality with a 1% rise GDP causing a change in the rate of child mortality (0.3–0.8%). Weil (9) shows that economic growth is highly correlated with health both across and within countries. Sharma (10) studied the health-growth relationship using the generalized moments-estimator method for 17 advanced economies from 1870 to 2013, showing a positive relationship between physical wellness and income. Based on the data collected from 134 developing countries during the period from 1970 to 2015, Cole (11) reported economic-growth effects on welfare, as economic advancements decreased child fatality rates while also raising life expectancy and caloric intake. He also found that economic growth reduced as national income increased. On the other hand, Sirag et al. (33) used a dynamic panel threshold model with 112 developed and developing countries from 1981 to 2010. Findings indicate the existence of a non-linear relationship between life expectancy (longevity) and economic growth. Longevity is useful for economic growth but only up to a certain level; any further increase in longevity above the threshold adversely affects growth. These findings emphasize the role of demographic transitions in explaining the relationship between health and economic growth.

Tobacco and alcohol consumption increase with income levels (9). Moreover, personal habits, such as overconsumption and an inactive lifestyle cause affluent diseases such as obesity. Individuals may not have sufficient personal time to invest in better health due to job-related pressures during economic growth periods (20). Thus, various studies show a pro-cyclical relationship between economic advancement and mortality, while a counter-cyclical pattern, economic growth and health improvement is also documented, e.g., Ruhm (12, 13), Economou et al. (15),Cutler et al. (34), Van den Berg et al. (17).

Ruhm (13) reported that a 1% reduction in professional layoffs was forecasted to cause a rise in heart disease by 0.75% in the United States, from 1979 to 1998. Coronary heart disease mortality rises rapidly when an economy is growing but returns to normal within 5-years. In contrast, Economou et al. (15) showed that a significant linkage exists between negative societal conditions and fatality in a sample from 13 EU countries. Granados (16) reported that mortality rates in postwar Japan tended to rise during economic expansion and dropped during contractions. Van den Berg et al. (17) reviewed the link between fatality and business cycles (individual as well as county-level) *via* the entire male population of Sweden, aged between 20 and 64 from 1993 to 2007, showing a pro-cyclical relationship between the business cycle and fatality levels. Cutler et al. (34) described how the aftermath of a strong business cycle varies due to an increased revenue impact on physical wellness and a negative influence from increased workload.

Researchers have found contrary evidence, e.g., economic recession is pro-cyclical for health–health improvements during economic downturns. Dehejia and Lleras-Muney (18) found that children's health improved during economic downturns for the United States from 1975 to 2000. Neumayer (19) studied the influence of government unemployment and commercial development statistics on fatalities in Germany from 1980 to 2000, showing lower fatalities during times of deflation.

Studies such as (20–24) show a variable link between health and business cycles with a counter-cyclical linkage to longevity and pro-cyclicality of life span. Ruhm (20) reports that the disadvantageous health effects from economic upturns can be prolonged. As an economy expands, smoking, drinking, and obesity levels rise while physical activity decreases. Brenner (21) shows that economic growth over varied time periods is correlated to mortality rates in the US during the 20th century. Expansion of industrialization and traffic increases injury-related mortality, lowers immunity levels, increases tobacco, alcohol, and saturated fat consumption. Granados and Ionides (23) revealed that economic expansion is linked with physical progress in Sweden in the 19th century but that relationship turns less positive with time and reverses in the latter years of the 20th century. Ruhm (35) showed severe-recession effects in the United States between 1976 and 2010, where the aggregate mortality changed from being very pro-cyclical to mildly unconnected to economic conditions. The relationship may be inaccurately measured at short timeframes (<15 years). Using a continuous wavelet analysis, Chen (24) examined the dynamic connection between health progress and economic growth from 1934 to 2010, in the US, and resolved the counter-cyclicality of longevity in the short term with the pro-cyclicality of life expectancy longer business cycles. Cole (11) implied that economic growth is pro-cyclical for health, but if the society is richer, the economic effect on health becomes weaker.

In contrast, Barro et al. (29) points out that life expectancy is a significant contributor to economic development. Bloom and Canning (36) document health improvements with labor market participation. Various studies such as (2, 3, 36–42) imply that longevity contributes to commercial development. Aghion et al. (39) indicate that long life expectancy encourages growth with healthier individuals who adjust to newer technologies. Swift (40) set up a co-integration linkage amongst life expectancy and GDP in 13 OECD countries for two centuries showing a 1% rise caused a 6% rise in GDP. Jones and Klenow (41) used consumption, leisure, mortality, and inequality to evaluate a country's economic well-being, showing that deviations are large where welfare is highly related to GDP. Siddique et al. (42), using fixed and random effect approaches, reviewed the role of education and health in terms of economic advancements in 76 middle-income countries, from 1991 to 2016, showing a link between longevity and economic expansion, while the opposite is true for child mortality. These studies have not been supported by research from Soares (43) who argues that economic expansion and longevity are now progressively disjointed, especially in numerous underprivileged countries around the world.

From the above review, it is clear that the longevity-growth nexus has been the subject of a series of debates. Many studies indicate that a higher life expectancy positively affects economic growth (44–49). On the other hand, various empirical studies point out that the relationship between longevity and economic growth might exhibit a non-linear pattern, especially when demographic changes are taken into account (50). The main purpose of our study is to re-investigate the relationship between life expectancy and economic growth using data from both China and India over the period between 1992 and 2015—two countries with the world's biggest populations. We will investigate how the longevity-growth pattern in these two countries compare.

## Data and Methodology

### Data

We employed a Bootstrap ARDL test developed by McNown et al. (28) to research the long-term link and Granger causality among economic growth, longevity (LE), and alcohol consumption (ALC). Yearly data from the World Bank was used to understand if alcohol consumption, economic growth, and longevity are related to economic growth in India and China. Gross domestic product (GDP) represents economic growth. The periods of study cover 1992 to 2015, but sample periods begin and end at different periods (different sample sizes) for different countries. Table 1 shows the data series summary, with China clearly surpassing India in terms of economic growth, longevity, and alcohol consumption. Skewness tests indicate that China's alcohol consumption data is skewed to the left while India's is skewed to the right.

**Table 1:** Data description.

	**GDP_CHINA**	**GDP_INDIA**	**LE_CHINA**	**LE_INDIA**	**ALC_CHINA**	**ALC_INDIA**
Mean	10.03691	12.04301	73.72632	64.93684	4.421053	1.994737
Median	9.996242	12.02428	74.10000	64.90000	4.900000	1.900000
Maximum	11.14049	12.72613	76.00000	68.40000	5.800000	3.100000
Minimum	8.983629	11.43379	70.60000	61.40000	2.900000	0.900000
Skewness	0.060023	0.154473	−0.448266	−0.008901	−0.133630	0.177633
Kurtosis	1.550272	1.694879	1.936737	1.801810	1.325313	1.504744
Jarque-Bera	1.675264	1.424040	1.531319	1.136814	2.276837	1.869921

#### Methodology

This article applies a Bootstrap Autoregressive Distributed Lag (ARDL) test described by McNown et al. (28). This technique is based on the ARDL cointegration test developed by Pesaran et al. (51).

#### ARDL Bound Test (51)

In general, a 2-variable ARDL based on Pesaran et al. (51) can be written as follows:

(3) $$\begin{array}{l} y_{t}=\;c+\;\emptyset y_{t-1}+\gamma x_{t-1}+\sum_{i=1}^{p-1}\lambda_{i\;}\Delta y_{t-i}+\sum_{j=1}^{q-1}\delta_{j}\Delta x_{t-j}\\ \;+\sum_{l=1}^{s}\omega_{l}D_{t,l}+\varepsilon_{t} \end{array}$$

Equation (3) shows that Y to X are unrelated, illustrating that we did not permit more of the variables to be endogenous, causing it to subvert the preconceived distribution of the numbers illustrated by Pesaran et al. (51). The weaker exogeneity of the regressors that are not impacted by the dependent variable for a longer period of time fails to rule out co-integration in regression, not assuming there is no immediate relationship from dependent variables to regression. Many researchers tend to set aside the hypotheses in the statistical information of the ARDL bounds test. *F*-test or *t*-test exists in cointegration.

$$\begin{array}{l} H_{0\;}:\emptyset =\gamma =0\;or\;H_{0}:\emptyset =0 \end{array}$$

#### Bootstrap ARDL Test (28)

McNown et al. (28) made use of the bootstrap method with the co-integration ARDL test, reporting that the tests have an appropriate portion and adequate power 10 characteristics and suggesting that the existing *F*-test and *t*-test of co-integration proposed by Pesaran et al. (51) should be compensated by adding a —test. In order to separate and identify cointegration, non-cointegration, as well as degradation defined by Pesaran et al. (51), all tests must be used. By making use of the research done by McNown et al. (28), the degradation situation can be understood in the following manner:

- **Degenerate case #1** takes place when both the *F*-test and *t*-test on the lagged independent variables are important, but the *t*-test on the lagged dependent variable is not important.
- **Degenerate case #2** takes place if the *F*-test and *t*-test on the lagged dependent are important, but the lagged independent variables are insignificant. The benefits from conducting the ARDL test are the results that validate the Monte Carlo simulations of asymptotic critical values; endogenous problems have minimal influence on the impact and the capability on the ARDL test. Apart from that, the correct resampling method will also allow the tests to perform at a higher level of superiority than asymptotic tests since it considers power and size attributes.

#### Granger Causality Test Based on Bootstrap ARDL Model

We can extend the Equation (1) to a 3-variable case defined as the follows:

(4) $$\begin{array}{l} \Delta y_{t}=\emptyset y_{t-1}+\gamma x_{t-1}+\varphi z_{t-1}+\overset{p-1}{\underset{i=1}{\sum }}\lambda_{i\;}\Delta y_{t-i}+\overset{q-1}{\underset{j=1}{\sum }}\delta_{j}\Delta x_{t-j}\\ \;+\overset{r-1}{\underset{k=1}{\sum }}\pi_{k}z_{t-k}+\overset{s}{\underset{l=1}{\sum }}\omega_{l}D_{t,l}\;+\varepsilon_{t} \end{array}$$

In the short run, the Granger-causality test for x y should capture the differences that lag on x along with the level of lag of x to identify if γ > 0 and δ = 0. For z y, there ought to exist the differences of lag on z as well as the level of lag of z to understand if ϕ > 0 and π = 0 (under cointegration situation).

## Empirical Results and Policy Implications

Table 2 shows results for the Bootstrap ARDL test that do not require strict assumptions, allowing for modeling variables with various orders of integration permitting I (0) and I (1) time series in the long-term relationship without I (2) variables 1 (51). Consequently, the application of several traditional unit root tests is required, i.e., the Augmented Dickey-Fuller (ADF), Phillips and Perron (PP), and Kwiatkowki et al. (KPSS) to examine if each of the time-series variables are stationary. Evidence from these three tests are shown in Table 2, demonstrating that all three variables are either I (1) or I (0), satisfying the Bootstrap ARDL test's pre-requirements.

**Table 2:** Univariate unit root tests.

	**Level**			**First differences**		
	**ADF**	**PP**	**KPSS**	**ADF**	**PP**	**KPSS**
**GDP_CHINA**	−2.859^**^ [3]	0.152 [2]	0.579^**^ [3]	−1.470 [3]	−2.024 [6]	0.194 [2]
**LE_CHINA**	−5.69^***^ [0]	−4.713 [2]	0.580^**^ [2]	−2.027 [3]	−0.991 [2]	0.447^*^ [1]
**ALC_CHINA**	−1.088 [1]	−1.021 [2]	0.304[3]	−3.141^**^ [0]	−3.141^**^ [0]	0.291 [1]
**GDP_INDIA**	−3.060^*^ [3]	−0.243 [2]	0.579^*^[3]	−1.297 [3]	−1.971562 [5]	0.183 [2]
**LE_INDIA**	−0.951 [1]	−1.651 [8]	0.592^*^[3]	−5.445^***^ [0]	−5.803^***^ [4]	0.244 [6]
**ALC_INDIA**	−0.563 [0]	−0.611 [2]	0.411^*^[1]	−4.337^***^ [0]	−4.333^***^ [1]	0.255 [1]

***, **, and * *indicate the null hypothesis is rejected at the 1, 5 and 10% levels, respectively. The number in brackets indicates the lag order selected based on Schwarz information criterion. The number in the parenthesis indicates the truncation for the Bartlett Kernel, as suggested by the Newey-West test (1987)*.

Table 3 documents the conclusions drawn from our Bootstrap ARDL cointegration tests. Results show that we can eliminate the null hypothesis of all *F*-tests and *t*-tests for China and India in the GDP equation. For both countries, GDP serves as the dependent variable, while longevity and alcohol consumption are explanatory. Namely, longevity and alcohol consumption account for the commercial growth of both countries for the longest time. Whereas, the dependent degenerate is rejected for China and lagged, while the independent *F*-test and variable F_dep_ are unimportant, so the degenerate case #2 is not sufficient to set up the code in the long run. In other words, all three-test statistics, *F*-test, *F*_indep_, and *T*_dep_ must be significant, and we find this to be the case in the China and the GDP equation.

**Table 3:** Cointegration results using bootstrap ARDL bound test.

	**DV|IV**	**Dummy variable**	***F***	F*_	***T*_**dep**_**	T^*^_dep_	***F*_**indep**_**	F^*^_indep_	**Result**
**GDP_CHINA**	GDP| alc,le	d03d07d11	6.730	2.917	−4.223	−1.203	10.020	3.228	Cointegration
**LE_CHINA**	LE|gdp,alc	d95d00d06d9	4.278	3.058	−1.490	−1.003	1.014	3.412	Degenerate #2
**ALC_CHINA**	ALC|gdp,le	d98d02d07	2.977	3.083	−0.439	−1.809	2.963	3.365	NO- cointegration
**GDP_INDIA**	GDP| alc,le	d03d07d11	8.267	4.278	−4.795	−2.651	12.186	4.603	Cointegration
**LE_INDIA**	LE|gdp,alc	d00d06d09	3.264	3.772	−1.020	−1.678	1.795	3.980	NO- cointegration
**ALC_INDIA**	ALC|gdp,le	d02d07d11	2.977	3.083	−0.439	−1.809	2.963	3.365	NO- cointegration

[.] is an optimal lag order based on Akaike Information Criterion (AIC). F is the F-statistic for the coefficients of yt−1, x t−1, and zt−1; Tdep denotes the t-statistics for the dependent variable, Tindep denotes the t-statistics for the independent variable. F^*^, T_dep, and T_indep are the critical values at 5% significance level, generated from the bootstrap program. Dummy variables are to capture any economics shocks. D03 means 1 for the year 2003, other years are 0.

Table 4 shows evidence derived from the Granger causality test results, reported as follows:

**Table 4:** ARDL Granger-causality analysis.

**Long-term**				
**Country**		**Δgdp equation**		
		*F* or *t* statistic (*p*-value) *F* or *t* statistic (*p* value) *F* or *t* statistic (*p* value)		
**China**	Δ gdp_t_, *gdp*_*t*−1_,	n.a.		
	Δ life_t_, *life*_*t*−1_	2.6095 (0.381) (–)		
	Δ alc_t_ *alc*_*t*−1_ *gini*_*t*−1_,	0.7498 (0.403) (–)		
**India**	Δgdp_t_, *gdp*_*t*−1_,	**n.a**.		
	Δlife_t_, *life*_*t*−1_	**0.0544 (0.299) (−)**		
	Δalc_t_, *alc*_*t*−1_*gini*_*t*−1_,	2.8023^*^ **(0.059) (−)**		
**Short-term**				
**Country**		Δ*gdp equation*	Δ*life equation*	Δ*gini alc equation*
		*F* or *t* statistic (*p* value) *F* or *t* statistic (*p* value) *F* or *t* statistic (*p* value)		
**China**	Δ gdp_t_, *gdp*_*t*−1_,	n.a.	7.207^**^ (0.019) (–)	1.9212 (0.3854) (–) n.a.
	Δ life_t_, *life*_*t*−1_	0.2763 (0.681) (+)	n.a	41.263^***^ (0.005) (–)
	Δ alc *alc*_*t*−1_ *gini*_*t*−1_,	1.9556 (0.180) (+)	1.518 (0.241) (+)	n.a
**India**	Δgdp_t_, *gdp*_*t*−1_,	**n.a**.	0.1586 (0.290)(-)	1.1001 (0.231) (+)
	Δlife_t_, *life*_*t*−1_	**0.02479 (0.839) (−)**	n.a.	17.442^***^ (0.005) (+)
	Δalc_t_, *alc*_*t*−1_*gini*_*t*−1_,	**0.02037 (0.859) (**–**)**	246.909 ^***^(0.00 0) (+)	n.a.

*Value in [.] is lag order, and (.) are p-value and sign for the coefficients. Bold values refer to the case of cointegration and the causality test involved its lagged level and differenced variables. Those values not in bold refer to the case of no-cointegration and its causality test involved only lagged differenced variables*.

****, **, and * *denote significant at 1, 5 and 10% levels, respectively*.

(1) Results from Bootstrap ARDL Test–Cointegration Test

**Figure 1:**
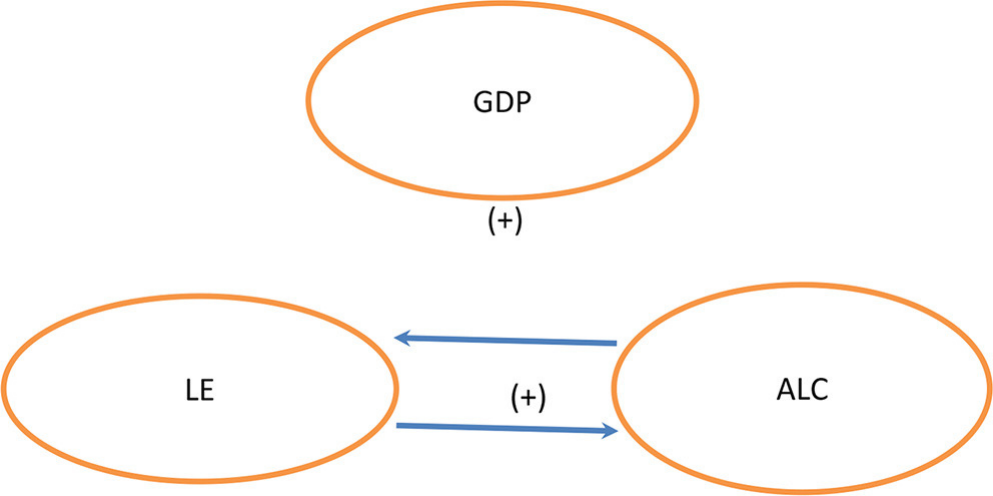
India.

**Figure 2:**
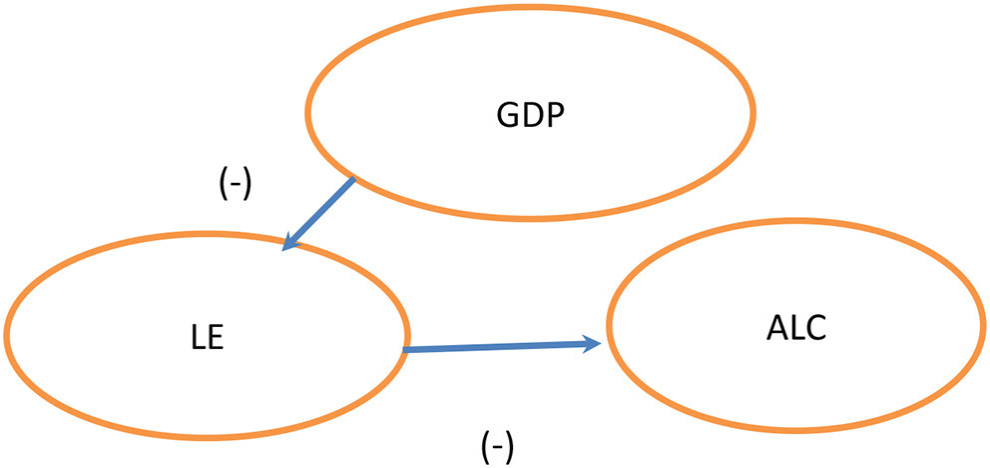
China.

Results of the Bootstrap ARDL cointegration tests show that longevity and alcohol consumption explain economic improvements in India and China in the coming few years. As Figure 1 shows, a dual-directional positive relationship exists between longevity and alcohol consumption (52), but longevity and alcohol consumption are not related to economic expansion. This shows a change in values of the younger generation. According to the World Health Organization (WHO), India's per capita alcohol consumption reached 5.7 L in 2016, about 2.4 times the level in 2005. However, global consumption per person rose by only 16% in 2005. Our empirical results are consistent with those of Alcohol org. (52). However, our results do not support those of Weil (9) and Kefeli and Abdul Azeez (30), in which they considered longevity and alcohol consumption to be related. In China, we see a unidirectional Granger causality linking longevity to economic growth and longevity to consumption of alcohol. The independent variables are negatively related to economic growth (20). Our findings do not aligning with those of Arora (37), Becker et al. (3), Bloom and Canning (36), Murphy and Topel (38), Aghion et al. (39), Swift (40), Jones and Klenow (41), Siddique et al. (42), and Gallardo-Albarrán (2), who all assert that economic growth positively affects longevity. Empirically, life expectancy at birth also negatively and significantly affects alcohol consumption in China. However, alcohol consumption has no effect on economic growth in Figure 2.

(2) Granger Causality Test Results Based on Bootstrap ARDL Model and Policy Implications

From a long-term perspective, we found that a co-integration relationship exists between longevity and alcohol consumption within GDP equations for both China and India, suggesting that longevity and alcohol consumption affect economic growth. Developed economies and emerging economies exhibit a connection between GDP and longevity, with emerging countries showing a stronger relationship (47, 53). Manthey et al. (54) observed, “Economic growth seems to explain the global increase in alcohol use over the past few decades. For example, the economic transitions and increased wealth of several countries—in particular, the transitions of China and India—were accompanied by increased alcohol use.” In China, according to the study, 77% of the population consumed alcohol at least occasionally in 2017. The Chinese on average drank 7 liters of alcohol that year, a 70% increase from 1990. In comparison with China, people in India drank an average of 6 liters of alcohol per person, which is double the consumption of 1990. On the other hand, the short-term unit negative Granger causality relates to economic growth's relationship to longevity, suggesting that economic pressures reduce longevity (55). Longevity negatively affects alcohol consumption, suggesting that high longevity means low alcohol consumption (56). Since there is no link between commercial growth and alcohol use in the two countries, this implies that alcohol consumption fails to have any immediate effect on economics. Examining life expectancy at birth, we observe different effects from alcohol consumption in China and India. In China, longevity exhibits negative causality with alcohol consumption (57); in contrast, India exhibits positive causality, suggesting differences in demographics between China and India. As stated by the World Bank, the number of elderly people in China is larger, at 147.5 million, and accounts for about 22.533% of the number of elderly people in the world, while in India the number is 80.23 million, or 12.256% of the total world population's elderly. Elderly people in China thus outnumber India by 1.84 times. In terms of the young population demographic, India has 200 million more people than China's population under the age of 20, which is 60% more. In addition, the impact of globalization and economic liberalization affects attitudes toward alcohol consumption in India (58). Furthermore, drinking age has also decreased significantly (58). Based on empirical results, it is important for the Chinese government to pay attention to the aging population problem. However, since India's population structure is relatively young, the Indian government must lower the drinking age. To conclude, findings from our study illustrate crucial policy implications for the government of China, which can attempt to solve the aging population issue in order to sustain economic growth, while the Indian government needs to face the impact of a younger drinking age on longevity.

## Conclusions

The current study investigates the relationship across economic development, longevity, and alcohol use in India and China. Results show a unidirectional negative Granger causality related with both economic growth and longevity just as longevity and alcohol consumption are also negatively related in China. These results are consistent with Vaupel (59), Ruhm (12, 13), Economou et al. (15), Granados (16), Cutler et al. (34), Van den Berg (17), Jakovljevic et al. (60), and Jakovljevic et al. (61) who emphasize a counter-cyclical relationship between economic growth and longevity in China. As incomes increase, so does traffic and business activity, such as alcohol and saturated fat consumption, decreasing immunity levels, directly raising injury-related mortality and decreasing longevity. Thus, the policy implications from these findings, related to population health during economic expansion, are important and practical. China is quickly becoming an aging society, with the population of the elderly rising since 2000. This will have a deep economic impact on decreasing alcohol consumption in China. This may explain why longevity and alcohol consumption is negatively associated within China. However, the opposite is true for India, with a positive bidirectional causality between longevity and alcohol consumption. This finding is consistent with the studies of Mondal et al. (62), Nadkarni et al. (63), Australian Institute of Health and Welfare (64), and Ogura and Jakovljevic (65) who have shown a positive linkage of age with consumption of alcohol. Comparatively lower GDP indicates a higher level of poverty and a general feeling of insecurity, including employment and housing, which results in stress and an increasing demand for alcohol. Thus, India, the country with a lower average life expectancy, i.e., 68.4 years (52), also needs health-policy makers to spread awareness of the complicated connection between detrimental consumption of alcohol and health through increased mobilization of resources required to avoid the harms of alcohol consumption.

## Data Availability Statement

The datasets generated for this study are available on request to the corresponding author.

## Author Contributions

FL: topic, data collection, introduction, literature review, results discussion, reference, and manuscript review. M-CW: methodology, results, manuscript review and correction. All authors contributed to the article and approved the submitted version.

## Conflict of Interest

The authors declare that the research was conducted in the absence of any commercial or financial relationships that could be construed as a potential conflict of interest.
